# Inhibitory coupling between inhibitory interneurons in the spinal cord dorsal horn

**DOI:** 10.1186/1744-8069-5-24

**Published:** 2009-05-12

**Authors:** Charalampos Labrakakis, Louis-Etienne Lorenzo, Cyril Bories, Alfredo Ribeiro-da-Silva, Yves De Koninck

**Affiliations:** 1 Unité de neurobiologie cellulaire, Centre de Recherche Université Laval Robert-Giffard, Québec, Québec, Canada, G1J 2G3; 2 Department of Pharmacology and Therapeutics, McGill University, Montréal, Québec, Canada, H3G 1Y6; 3 Department of Anatomy & Cell Biology, McGill University, Montréal, Québec, Canada, H3A 2B2

## Abstract

Local inhibitory interneurons in the dorsal horn play an important role in the control of excitability at the segmental level and thus determine how nociceptive information is relayed to higher structures. Regulation of inhibitory interneuron activity may therefore have critical consequences on pain perception. Indeed, disinhibition of dorsal horn neuronal networks disrupts the balance between excitation and inhibition and is believed to be a key mechanism underlying different forms of pain hypersensitivity and chronic pain states. In this context, studying the source and the synaptic properties of the inhibitory inputs that the inhibitory interneurons receive is important in order to predict the impact of drug action at the network level. To address this, we studied inhibitory synaptic transmission in lamina II inhibitory interneurons identified under visual guidance in spinal slices taken from transgenic mice expressing enhanced green fluorescent protein (EGFP) under the control of the GAD promoter. The majority of these cells fired tonically to a long depolarizing current pulse. Monosynaptically evoked inhibitory postsynaptic currents (eIPSCs) in these cells were mediated by both GABA_A _and glycine receptors. Consistent with this, both GABA_A _and glycine receptor-mediated miniature IPSCs were recorded in all of the cells. These inhibitory inputs originated at least in part from local lamina II interneurons as verified by simultaneous recordings from pairs of EGFP+ cells. These synapses appeared to have low release probability and displayed potentiation and asynchronous release upon repeated activation. In summary, we report on a previously unexamined component of the dorsal horn circuitry that likely constitutes an essential element of the fine tuning of nociception.

## Findings

The superficial laminae of the dorsal horn act as a relay station for nociceptive sensory signals. They receive inputs from unmyelinated C- and thinly myelinated Aδ-fibers [1-3] and process this input through a network of local inhibitory and excitatory interneurons before relaying it to supraspinal centers. Inhibitory interneurons are key players in the regulation of spinal excitability, by releasing the neurotransmitter GABA and, in many cases, co-releasing glycine [4]. Their important role in pain processing is illustrated by the finding that local blockade of GABA_A _receptors (GABA_A_Rs) and glycine receptors (GlyRs) replicates symptoms of neuropathic and inflammatory pain [5-8]. Blocking this form of inhibition also allows low threshold input to be relayed to normally nociceptive specific neurons [9-12]. Despite this, very little is known about the functional circuitry of the dorsal horn and, in particular, on inhibitory connections onto GABAergic interneurons. This is an important aspect of the dorsal horn circuitry as inhibitory inputs onto inhibitory neurons would reduce their output leading to net disinhibition and spinal hyperexcitability.

**Figure 1:**
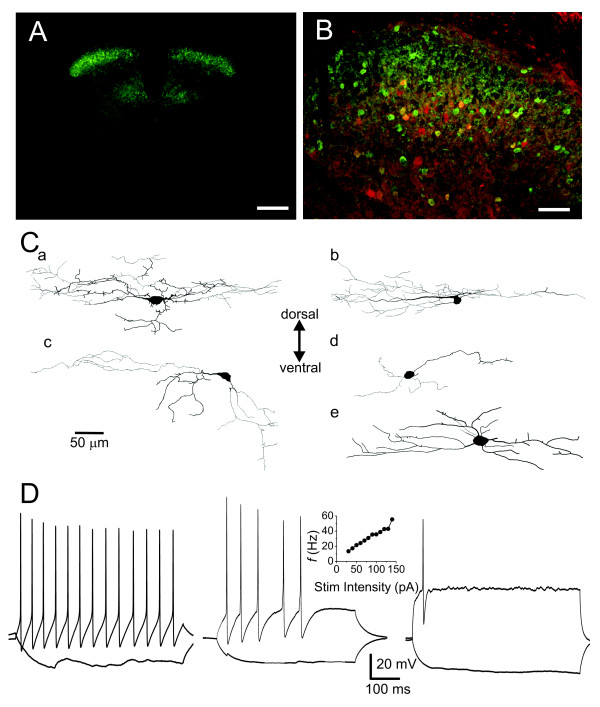
**Characterization of the GAD65-EGFP+ neurons**. A. Photomicrograph of a lumbar transverse section of the spinal cord showing the distribution of EGFP expression. Scale bar = 200 μm. B. immunostaining for parvalbumin (*red*) and GFP (*green*) shows some co-localization in lamina II (*yellow*). Scale bar = 50 μm. C. Examples of EGFP+ neurons, reconstructed from neurobiotin/Lucifer Yellow filled cells. *a-b*: islet cells, *c*: vertical cell, *d-e*: unclassified cells. All cells are shown in the parasagittal plane. The majority of the dye filled cells were islet cells (9/15), one cell was vertical and the rest (5/15) could not be classified. D. Firing patterns in response to 500 ms-long depolarizing and hyperpolarizing current steps from a holding potential of -65 mV. *Left*: The response of a tonic firing neuron to current steps of -30 pA and +40 pA. *Middle*: A neuron that fired one or few spikes at the onset of the depolarization. Responses to current steps of -30 pA and +40 pA. The *Inset *shows the average instantaneous firing frequency (*f*) plotted against stimulus intensity (I). The *f*-I curve for this neuron being linear, it was classified as tonic firing according to previously established criteria [20]. *Right*: Response of a single spiking neuron to -30 pA and +90 pA current steps.

Towards this, we used transgenic mice that express EGFP under the control of the glutamate decarboxylase 65 gene (GAD65), the enzyme that catalyzes the production of GABA[13], to be able to visually identify GABAergic interneurons and perform targeted recordings from them in live spinal cord slices. Figure 1A shows a high density of EGFP+ neurons in the superficial laminae as well as in the medial part of lamina V in these mice. Previous reports indicate that, in this transgenic line, virtually all (> 98%) of EGFP+ cells co-express GAD65 [14]. In addition, we found that 25% of the EGFP+ cells co-expressed parvalbumin (Figure 1B), consistent with findings in another mouse line [15]. Previous studies report that parvalbumin-immunoreactive GABAergic cells also express glycine [16], indicating that a subpopulation of EGFP+ cells in our study are also glycinergic. EGFP+ neuron morphology (Figure 1C) was comparable to that previously described for inhibitory interneurons in mice [15,17] and rats [18,19].

We then studied the firing responses of the cells to depolarizing pulses as previously described to further characterize the population [15,17,19-22]. Of the 33 neurons tested, 23 showed a clear tonic firing pattern (Figure 1D). An additional 4 cells showed a firing pattern that could be interpreted, at first sight, as phasic or initial bursting [15,20] (Figure 1D). Using isolated responses can however lead to misclassification. Indeed, in a previous study, we showed that the decisive classification criterion is the nature of the input-output curve of the cell: tonic cells display a characteristic linear encoding curve, while phasic cells encode non-linearly with an abrupt increase in the *f*-I curve at low stimulus intensity [20], yielding a discontinuous *f*-I. The latter difference in encoding properties of tonic vs. phasic neurons corresponds to class 1 vs. class 2 excitability as defined by Hodgkin's initial classification [23]. Using this classification criterion, we found that these 4 additional cells fell under the tonic class (Figure 1D). Previous work also suggests identity of the two populations based on the ability of individual neurons to convert from one pattern to the other [24]. The remaining 6 neurons had a single spike firing pattern (Figure 1D). These data are consistent with previous reports on dorsal horn inhibitory interneurons in mice [17,22,25] and rats [3,18].

**Figure 2:**
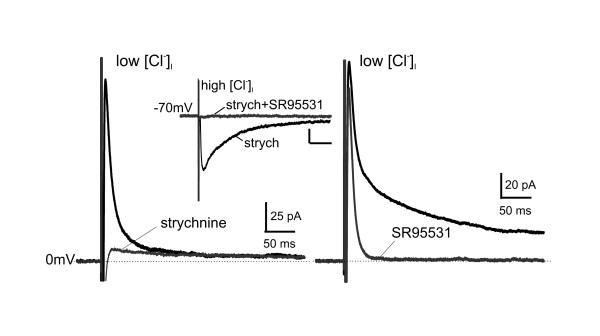
**Lamina II inhibitory neurons receive both GABA_A_R- and glycine receptor-mediated inhibitory inputs**. IPSCs evoked by focal electrical stimulation while recording at a holding potential (HP) of 0 mV using CsMeSO_3_-filled micropipettes (low [Cl^-^]_i_). The traces represent averages of 10 consecutive responses. These IPSCs displayed complex kinetics. Administration of strychnine (0.5 μM; *Left*) blocked the fast decaying component, indicating that it was mediated by activation of glycine receptors, while administration of SR95531 (gabazine, 10 μM; *Right*) blocked the slow decaying component, indicating that is was mediated by activation of GABA_A _receptors. The relative contribution of the two components varied from cell to cell: the fast component comprised 78% (range 54%–94%) of the peak, while the slow component 22% (range 6%–46%) *Inset: *IPSCs evoked by focal stimulation while recording at a holding potential of -70 mV using CsCl-filled micropipettes (high [Cl^-^]_i_) to record inward currents (Scale bars = 25 pA, 50 ms). Recordings were performed in the presence of strychnine and addition of SR95531 abolished all remaining inward currents indicating that the evoked responses were entirely mediated by GABA_A _and glycine receptors.

To test for inhibitory input to GAD65-EGFP+ neurons, we recorded responses to focal stimulation in the presence of CNQX and APV. Under these conditions, monosynaptic eIPSCs with complex decay kinetics were recorded in all cells: a fast component (12.6 ± 0.5 ms) and a slower component (200.8 ± 26.9 ms) (n = 11; Figure 2). The fast component was blocked by the GlyR antagonist strychnine (0.5 μM; n = 8; Figure 2) and the slow one by the GABA_A_R antagonist SR95531 (10 μM; n = 6; Figure 2). Complex eIPSC are the result of activation and release from several GABAergic and glycinergic axon terminals and thus complex GABA_A_R and GlyR mediated eIPSCs should not be misinterpreted as evidence of co-release of the two neurotransmitters from the same terminal.

**Figure 3:**
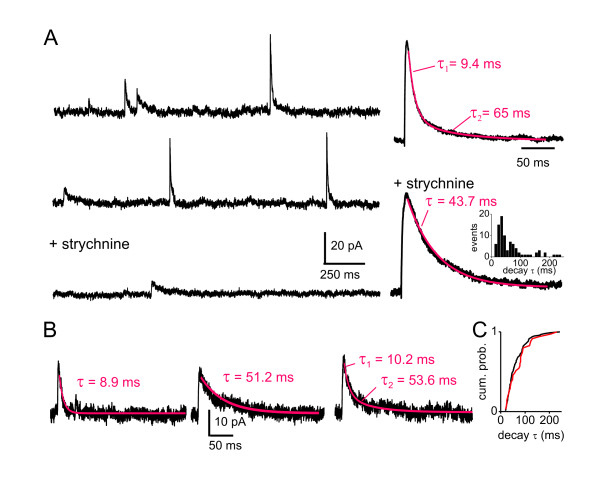
**GABA_A_R- and glycine receptor-mediated mIPSCs in Lamina II inhibitory neurons**. A. Recordings of mIPSCs in presence of 1 μM TTX at a holding potential of 0 mV using CsMeSO_3_-filled micropipettes. mIPSC frequency was 0.38 ± 0.06 Hz (n = 9). The traces on the *left *show mIPSCs in control conditions (*top *and *middle*), displaying different decay kinetics, and in the presence of strychnine (*bottom*) displaying only slow decay kinetics. The traces on the *right *are averages of 100 consecutive mIPSCs in control (*top*) and in the presence of strychnine (*bottom*). Administration of strychnine abolished the fastest component of the complex decay kinetics of the average mIPSCs confirming that this component was mediated by glycine receptors as previously established [26,27]. *Inset*: Histogram showing the distribution of GABA_A_R-mediated mIPSC decay τ in the presence of strychnine. B. Three individual mIPSCs showing characteristic different decay kinetics: fast decay (*left*; absent in strychnine thus presumably glycinergic); slow decay (*middle*; GABA_A_-mediated); mixed fast and slow (both glycine and GABA_A_-mediated). The proportion of fast, slow or mixed mIPSCs varied across cells. Fast decaying mIPSC proportions ranged from 17% to 93%, slow decaying mIPSCs from 5% to 78% and mixed mIPSCs from 1% to 10%. Five out of nine neurons showed predominately (> 70%) fast mIPSCs, two out of nine were having predominately (> 70%) slow mIPSCs. C. Cumulative probability plot of the slow mIPSC decay τ (*black*) and the decay τ of the slow component from mixed mIPSCs (*red*), no significant difference between the two populations was found (p > 0.05; Kolmogorov-Smirnov test). All recordings we performed in the presence of 10 μM CNQX and 40 μM APV.

To test whether IPSCs at individual synapses had similar properties, we studied the decay kinetics of mIPSCs (Figure 3A). Average mIPSCs displayed similar dual decay kinetics: τ_1 _= 12 ± 0.9 ms and τ_2 _= 156.8 ± 64.4 ms (n = 9). As for eIPSCs, strychnine administration confirmed the fast component was mediated by GlyRs (n = 6). Individual mIPSCs could be distinguished on the basis of their decay kinetics (Figure 3B): mIPSCs with monotonic fast decay kinetics made up the majority of events (66%). These were absent in strychnine confirming that they were mediated by GlyRs only. Another subpopulation was characterized by slow decay kinetics (27%), consistent with them being mediated by GABA_A_Rs [19,26-28]. A remaining small subset of events showed a dual decay kinetics (7%) composed by a fast (< 14 ms) and a slower component (Figure 3C); they were absent in the presence of strychnine, indicating that they corresponded to dual GABA_A_R- and GlyR-mediated mIPSCs, consistent with co-release from the same synapse [26]. Additional experiments using pipettes filled with high Cl^- ^solutions to yield inward Cl^- ^currents at a holding potential of -70 mV showed similar kinetics. Under these conditions, all inward currents were abolished by addition of strychnine and SR95531 indicating that the evoked responses were entirely mediated by GABA_A_Rs and GlyRs.

**Figure 4:**
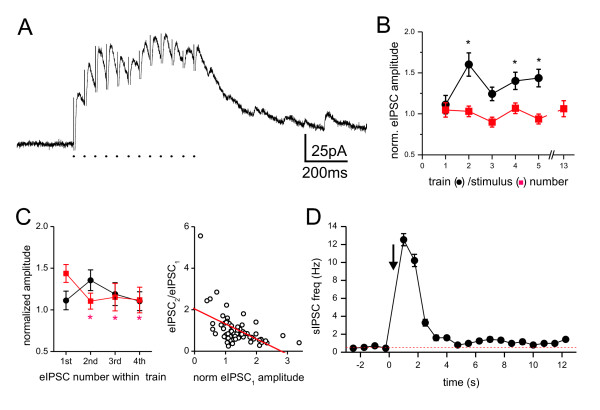
**Different forms of plasticity at inhibitory synapse onto lamina II inhibitory neurons**. Recordings were made in the presence of APV, CNQX and strychnine. A. Summation of evoked IPSCs (eIPSCs) caused by a train of 12 focal stimuli (*dots at bottom*) at 20 Hz. Stimulus artefacts are partially blanked. Note the occurrence of numerous sIPSCs at the end of the train indicative of asynchronous release. B. The normalized amplitude of the first eIPSC evoked by each train is plotted for the initial five consecutive repetitions of the trains (at 0.05 Hz; *black*). The amplitude of the first eIPSC in each train was normalized to the average of five IPSCs evoked by single stimuli (at 0.1 Hz) that preceded the train stimulation protocol. The values plotted are the means ± SEM from 20 neurons. The graph shows a significant (p < 0.05, Friedman test) increase in the eIPSC amplitude after the initial train (train number 1). Asterisks denote significant differences in subsequent values from the initial train (train number 1; Student-Newman-Keuls posthoc test; p < 0.05). For comparison, the normalized amplitudes of five consecutive IPSCs and the 13^th ^IPSC evoked by single stimuli are plotted (*red*). This indicates that the potentiation of the eIPSCs after the first train (of 12 stimuli) does not occur with accumulation of the same number of stimuli at low frequency. C. *Left*: Normalized amplitudes of the initial four eIPSCs within the first (*black*) and fifth (*red*) train. The values plotted are the means ± SEM from 20 neurons. *Right*: Ratios between the second eIPSC and the first eIPSC (eIPSC_2_/eIPSC_1_) from individual trains are plotted against the normalized amplitude of the first eIPSC. The data shows negative correlation between these values (linear fit: r = -0.53, p < 0.05), indicating depression of the second eIPSC when the first was potentiated and vice versa. D. Peristimulus time histogram showing the change in sIPSC frequency before and after each train (arrow; n = 15 trains from one cell).

To study the behaviour of this inhibitory synapse in response to repetitive activation, we recorded eIPSCs following trains of focal stimuli (Figure 4A). Using this paradigm, three forms of plasticity were observed. First, repetitive train stimulation caused a significant increase in amplitude of the first eIPSC of each train (Figure 4B; p < 0.05, Friedman test, n = 20 cells). Second, within individual trains, short term plastic changes followed opposite trends, depending on whether the first eIPSC of the train was potentiated or not; there was facilitation when the first eIPSC was not-potentiated vs. depression when the first eIPSC was potentiated (Figure 4C; n = 20). Third, in 16 out of 20 cells asynchronous release occurred at the end of each train of stimuli (Figure 4D). This is shown by the observation that the frequency of spontaneous IPSCs changed from 0.5 ± 0.08 Hz during the second preceding the train to 2.9 ± 0.4 during the second following the end of the train (paired t-test, p < 0.05, n = 20). Such asynchronous release phenomenon was observed for both pharmacologically isolated GABA_A_R and GlyR-mediated IPSCs. These specific plastic properties have been observed at other, but not all inhibitory synapses in the CNS [29-32].

**Figure 5:**
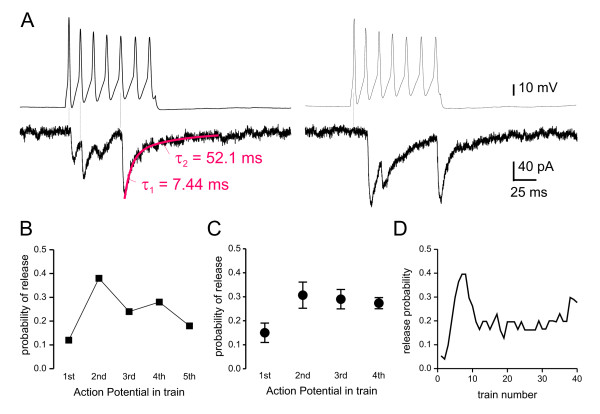
**Inhibitory coupling between pairs of lamina II inhibitory interneurons**. A. Example recordings from a pair of synaptically connected neurons. Top traces are from the presynaptic neuron showing a train of action potentials evoked by a 100 ms depolarizing current step. Bottom traces show simultaneous recordings of IPSCs from the postsynaptic cell. Note on the *right*, the release failure in response to the first action potential. Most IPSCs in this pair displayed fast decay kinetics with a slow component also visible in some events. B. Graph showing the probability *p *of release for each of the first five presynaptic action potentials in the train calculated from the pair of neurons shown in A (*p *defined as number of IPSCs per number of corresponding action potential in 50 consecutive trains delivered at 0.1 Hz). Note the dramatic increase in release probability in response to the 2nd action potential in the train. C. Probability of release for each of the first 4 action potentials in the train averaged for all 3 synaptically connected pairs studied. Values are means ± SEM. D. The probability *p *of release per train calculated for each train from the pair of neurons shown in A. The plot was smoothed with a running average of four values. Note the transient increase in probability after the initial trains.

The sources of the inhibitory inputs to inhibitory interneurons in lamina II could be other local interneurons, lamina III inhibitory interneurons [19,22] and/or descending fibres from the brain [33]. To test whether local inhibitory interneurons can themselves be the source of the IPSCs recorded on inhibitory interneurons, we performed simultaneous whole-cell recordings from pairs of EGFP+ lamina II neurons. In recordings from 56 EGFP+ pairs, 3 pairs were found synaptically connected. In 2 of the 3 pairs, the direction of the synaptic connection was tested and was found unidirectional in both pairs. The latency between the peak of the action potential and the onset of the IPSC ranged between 1.6 and 3.4 ms (mean 2.3 ± 0.5 ms). In one of the pairs, some IPSCs had clear dual decay kinetics (Figure 5A), with a fast component comparable to that mediated by glycine receptors as described above indicative of mixed glycine- and GABA_A _IPSCs. In the other two pairs, IPSCs were lacking the fast decaying component, indicating GABA_A_-only IPSCs. All three synaptically coupled pairs displayed high degree of failure. Probability of release in response to the first action potential of the train ranged between 0.12–0.23 (Figure 5B). In 2 of the pairs, probability of release increased significantly in response to the subsequent action potentials in the train (χ^2^, p < 0.05). In the third pair, the probability of release was not different in response to the first three action potentials but increased later (Figure 5B–D). Thus, the connection between dorsal horn neurons is very selective as shown by the rarity of synaptically connected pairs in this and previous studies [34]. In addition, such connectivity between inhibitory interneurons appears to display low fidelity (< 25%), in contrast to connections between inhibitory islet cells and excitatory transient central cells which showed high fidelity (60–100%) [18,34]. This suggests that our recordings are from different synapses from that previously described and thus consist in a novel component of the superficial dorsal horn circuitry.

Our results indicate that inhibitory interneurons impose inhibition locally on other inhibitory interneurons in the superficial dorsal horn. This synaptic arrangement can be the substrate of local disinhibition, which may play a key role in sensory processing. Indeed, GABAergic interneurons intercalated within the circuitry linking primary afferents and spinal projection neurons [25] may play a dual role in controlling network excitability: lamina II inhibitory interneurons can act, directly, to repress polysynaptic excitatory relay connections [11] or, indirectly, to potentiate these connections by silencing specific inhibitory components of the dorsal horn circuitry. Understanding how drugs may selectively affect such distinct inhibitory synapses is critical to predict the net result of a specific treatment on spinal output. Equally, the finding that inhibitory synapses impinging on inhibitory interneurons are characterized by specific properties opens the door for differential modulation of inhibitory vs. "disinhibitory" components of the spinal dorsal horn, yielding potentially more effective interventions.

## Methods

All experiments were performed in accordance with regulations of the Canadian Council and Animal Care. Mice in this study were heterozygous C57BL/6 BAC transgenic mice expressing enhanced green fluorescent protein (EGFP) under the control of the GAD65 promoter [14,35]. Adult transgenic mice (3–5 months) were anaesthetized with ketamine/xylazine and perfused intracardially with ice-cold oxygenated (95% O_2_, 5% CO2) sucrose substituted ACSF containing (mM) 252 sucrose, 2.5 KCl, 1.5 CaCl_2_, 6 MgCl_2_, 10 glucose, 26 NaHCO_3_, 1.25 NaH_2_PO_4 _and 5 kynurenic acid. Mice were decapitated, the spinal cord was removed by hydraulic extrusion and 250 μm thick parasagittal slices were cut from the lumbar portion [36]. Slices were kept in normal oxygenated ACSF (126 NaCl, 2.5 KCl, 2 CaCl_2_, 2 MgCl_2_, 10 glucose, 26 NaHCO_3_, 1.25 NaH_2_PO_4_) at room temperature until recording.

Slices were transferred in the recording chamber and continuously superfused at 2–3 ml/min with oxygenated ACSF at room temperature. Patch pipettes (7–8 MΩ) were filled with (in mM) 135 KCl, 10 HEPES, 2 MgCl_2_, 0.5 EGTA, 2 ATP, pH 7.2. For some recordings, KCl was substituted by 130 CsMeSO_3_/5 CsCl or by 135 CsCl. Whole cell patch clamp recordings were made using a Multiclamp 700B amplifier (Molecular Devices, Sunnyvale, California, UK). Data were low pass filtered at 10 kHz, digitized at 20–30 kHz and acquired with the Strathclyde electrophysiology software (WinWCP and WinEDR courtesy of Dr. J. Dempster, University of Strathclyde, Glasgow, UK).

Epifluorescence was used to identify EGFP+ neurons. All recordings in this study are from neurons with their somata located in lamina II. Action potential firing was analysed as previously described [20]. Only cells with resting membrane potential more negative than -50 mV were included in the study. Instantaneous firing frequency (*f*) was calculated as the reciprocal of the interspike interval.

The mIPSCs were detected and analysed using Mini Analysis (Synaptosoft, Decatur, Georgia, UK) and a locally designed software (YDK). Decay time constants were fitted using automated least square algorithms. The necessity to introduce additional exponential components to the fits was first judged on the basis of visual inspection. When the merit of additional components was not obvious, further statistical analysis was applied as previously described [26]. To simplify analysis, complex mIPSC that did not contain the strychnine sensitive, fast decay component (< 14 ms; i.e. mIPSCs that consisted of multiple GABA_A_R-mediated decay components), were treated as monotonic.

Monosynaptic IPSCs were evoked focally by electrical stimulation (30–70 μA, 250 μs) via patch pipette filled with ACSF placed 50–100 μm from the soma of the recorded cell as described previously [26]. Single stimuli or trains of stimuli (12 pulses at 20 Hz intraburst frequency) were delivered every 10 or 20 s, respectively. To isolate the complex decay components of the eIPSCs pharmacologically, all tested cells were treated with strychnine or SR95531. In all cases strychnine blocked only the fast component. Similarly, SR95531, blocked only the slow component in all cases. The relative contribution of each component to the peak current was calculated from the pharmacologically blocked portion (by subtraction) and the portion that was not blocked. In some experiments, strychnine and SR95531 were applied together and abolished all components of the evoked current. eIPSC amplitude measurements from train stimulations were measured from the point immediately before the stimulation artefact to take into account baseline changes due to IPSC summation.

In simultaneous recordings from pairs of neurons (located within < 250 μm of each other), one of the neurons, defined as presynaptic, was kept in current clamp mode and injected with a 100 ms-long depolarizing current pulse of 100 pA to elicit a train of 4–7 action potentials. The other cell, defined as postsynaptic, was held at -70 mV in voltage clamp mode and postsynaptic currents were recorded. These recordings were conducted using KCl filled pipettes to allow for action potential generation and maximum amplification of Cl^-^-mediated IPSCs. All glutamatergic transmission was blocked by APV and CNQX, thus all synaptic connections were assumed to be monosynaptic and inhibitory. This protocol was repeated for 40–50 trials at 10 s intervals for all tested pairs.

For immunostainings, animals were anaesthetized and perfused intracardially with 4% paraformaldehyde (PFA) in 0.1 M phosphate buffer. Spinal cord segments L4–L5 were collected, postfixed for 60 minutes in PFA and cryoprotected in 30% sucrose in phosphate buffer overnight at 4°C. Fifty μm-thick transverse sections were cut on a freezing microtome (Leica SM2000R). Sections were collected into tissue culture plates with 24 wells, washed with phosphate-buffered saline (pH = 7.4) and 0.2% Triton (PBST), and pre-treated with 5% donkey normal serum in PBST for 10 minutes.

The sections were washed twice in PBST and incubated overnight in a mixture of a mouse anti-parvalbumin antibody (1:4000, Sigma) and a rabbit anti-GFP antibody (1:500, Clontech Living Colors, Mountain View, CA) in PBST. After washing in PBST, the tissue was incubated for 2 hrs at room temperature in a Rhodamine Red-X-conjugated donkey anti-mouse IgG (H+L) antibody (1:500, Jackson, West Grove, PA) and in Alexa Fluor 488-conjugated IgG (H+L) donkey anti-rabbit antibody (Invitrogen, Carlsbad, California, USA) in PBST. Lastly, sections were washed for 15 minutes (3 × 5) with PBS, mounted on gelatin-subbed slides, allowed to dry overnight at 4°C and cover-slipped using Aquapolymount (Polysciences, Warrington, PA). The sections were observed with a Zeiss LSM 510 confocal microscope (Zeiss Canada). Images were obtained using a multi-track approach for the detection of two signals (Alexa Fluor 488 and Rhodamine Red-X), with the help of a 20 × water immersion objective. Data were collected with Metamorph 7 (Molecular Devices) from 1815 cells counted from 20 sections of three mice. Only cells with visible nuclei were counted.

For morphological classification of EGFP+ neurons, in a subset of electrophysiological experiments, neurons (n = 6) were recorded and filled with patch pipettes containing 0.5% neurobiotin. Slices were then fixed in 4% PFA in PB. In addition, 200 μm thick lumbar parasagittal slices taken from 4% PFA fixed spinal cords were used to inject EGFP+ neurons with Lucifer Yellow (LY) as described previously [37]. LY-injected slices were then incubated with a rabbit anti-LY antibody (1:20000) and subsequently with biotinylated goat anti-rabbit (1:500) antibody. Biotinylated antibodies and Neurobiotin were visualized with a horseradish peroxidase reaction (ABC kit, Vector Laboratories) and nickel-diaminobenzidine as a substrate. Cells were reconstructed using a light microscope with a computerized tracing system (Neurolucida, MicroBrightField Inc). Cells were classified according to previous descriptions [21]. As the distinction between islet and central cells is sometimes difficult [15] and due to our small sample, they were treated as one group.

Drugs were obtained as follows: 6-cyano-7-nitroquinoxaline-2,3-dione (CNQX) and D(-)-2-amino-5-phosphonopentanoic acid (D-APV) from Tocris Cookson (Ballwin, Montana, USa), Gabazine (SR95531) and strychnine hydrochloride from Sigma (St. Louis, Missouri, USA), and tetrodotoxin (TTX) from Alomone Labs (Jerusalem, Israel).

Data are presented as means ± SEM, n numbers refer to number of cells tested unless otherwise indicated. Normality of the data was tested with the Shapiro-Wilk test. Paired t-tests were used to compare IPSC frequencies. χ^2 ^tests for contingency tables were used to determine differences in release probabilities. Repeated measure comparison of non-parametric data were analysed using the non-parametric Friedman test and subsequently analysed with the Student-Newman-Keuls posthoc test.

## Competing interests

The authors declare that they have no competing interests.

## Authors' contributions

CL and YDK conceived and designed the study. CL performed the experiments, LEL performed the immunostainings, CB performed Lucifer Yellow injections and 2D reconstruction of EGFP+ neurons. All authors contributed to the preparation of the final manuscript.
